# Mental health care services in Nigeria: A qualitative enquiry from family physicians’ perspective

**DOI:** 10.1371/journal.pmen.0000285

**Published:** 2025-09-25

**Authors:** Olumide Thomas Adeleke, Akeem Opeyemi Akinbode, Omorovbiye Aibangbee, Eloho Joy Orji, Alarape Naomi Oluwasanya, Wulaimat Abimbolanle Adekunle, Oludaisi Adeshina Oduniyi, Aderemi Temitayo Olabode, Adekunle Joseph Ariba

**Affiliations:** 1 Family Medicine Department, Bowen University Teaching Hospital/Bowen University, Iwo, Osun, Nigeria; 2 Department of Family Medicine, Federal Teaching Hospital, Birnin, Nigeria; 3 General Hospital Otu Jeremi, Delta State, Nigeria; 4 Department of Family Medicine, Federal Medical Centre, Owo, Ondo State, Nigeria; 5 Department of Family Medicine, Olabisi Onabanjo University Teaching Hospital, Sagamu, Ogun State, Nigeria; 6 Centre for Family Medicine, Jericho Specialist Hospital, Ibadan, Oyo, Nigeria; 7 Department of Family Medicine, Lagos State University Teaching Hospital, Ikeja, Lagos, Nigeria; 8 Department of Public Health, Fountain University, Osogbo, Nigeria; 9 Department of Family Medicine, Olabisi Onabanjo University Teaching Hospital, Sagamu, Japan; University of Newcastle, AUSTRALIA

## Abstract

Mental health has emerged as a critical facet of human well-being, necessitating focused on global attention due to an exceptional rise in the prevalence of mental disorders worldwide. Nigeria, like many other countries, faces various mental health challenges that profoundly affect the lives of its populace. This is further aggravated by a near-total lack of integration of mental health services into the country’s existing health care structures. Therefore, this study aimed to assess the current situation of mental health services across Nigeria’s health systems from family physicians’ perspectives.This descriptive cross-sectional qualitative study that used an in-depth interview guide to collect data on the current status of mental health service provision in Nigeria among family physicians located in different levels of health care in all six geopolitical regions of the country. Interviews were conducted with 23 participants and analysed using content analysis and presented using thematic categorization. Five (5) themes emerged from the participants’ description of the current status of mental healthcare situation in Nigeria: (i) Stand-alone Health Facility and Coverage for Mental Health Service Provision in Nigeria, (ii) Inadequacy of Mental Health Specialists (iii) Increasing Prevalence and Burden of Mental Health in Nigeria, (iv) Poor knowledge of mental health management, and (v) Weak Policy on Mental Health Service. There are gaps to be filled to optimize mental health service delivery in Nigeria. The study participants identified five key factors that stakeholders need to consider improving the state of mental health services in Nigeria.

## Introduction

Mental health conditions (MHC) are among the leading causes of health loss globally, with 13.9% of the global population experiencing MHC in the year 2021 [1]. Mental health conditions account for 5.4% of the global disease burden [2]. According to the World Health Organization, up to 25% of the global population will experience a mental health condition at some point of their lifetime [3]. The Covid-19 pandemic of 2020 contributed to a rise of 18% more people experiencing depression and an increase of 13% more individuals developing anxiety disorders [1]. Mental health conditions account for 17.2% of the total life lived with disability worldwide [1]. Using the years lived with disability (YLDs) and disability-adjusted life years (DALY)measures,depressive disorders accounted for the second greatest cause of YLDs in 2021, showing a rise of 36.5% from 2010, while anxiety disorders constituted the sixth cause of YLD in the same year [2]. Between 2010 and 2021, the age-standardised DALYs rose by 16.4% for depressive disorders and 16.7% for anxiety disorders between 2010 and 2021 [2]. The other common MHC among the 25 highest causes of YLDs were alcohol use disorders, drug use disorders, dementias, schizophrenia, autism spectrum disorders, which accounted for 67.1 million YLDs combined [2].

In Nigeria, the prevalence of mental health conditions has been reported to be 11.1%. [1]. Approximately 40 million (20%) Nigerians are affected by mental health conditions [3]. In 2017, approximately 7 million (3.9%) and 4.9 million (2.7%) Nigerians had anxiety disorders and depressive disorders, respectively [3]. In low- and middle-income countries (LMIC) like Nigeria, up to 85% of people with mental, neurological, and substance use (MNS) disorders go untreated for their conditions [4]. Nigeria, like many other countries, has a diverse range of mental health challenges that significantly impact the lives of its citizens. Mental health is defined as the state of human wellbeing during which a person realises their own personal abilities, able to cope with routine life stresses, can work fruitfully and productively, and is capable of contributing to his or her community [5]. A poor level of awareness regarding mental health conditions in Nigeria [6]. Due to the societal perception of a spiritual or malevolent spirit as the cause of mental health conditions, most people seek treatment from spiritual healers rather than medical facilities [6].

### The health system structure in Nigeria

The healthcare system in Nigeria has faced various challenges over the decades since the country’s independence. The health system structure of the country is bdivided intothree levels, namely the primary, secondary, and tertiary levels. The primary health facilities are mainly within the local government authorities and with support from state governments. They are accessible majorly for people who dwell in rural settings, and care given is directed mainly at common health conditions, health promotion, and disease prevention activities. Mental health, although officially regarded as within the primary health care system of the country, is mainly excluded from the care provided at the level of either a lack of trained personnel or supportive structural health facilities. The secondary health level mainly comprises general hospitals and comprehensive health centres, which are mostly under the domains of the state governments. Most physician-led private hospitals also fall under this category as well. The tertiary health level comprises teaching hospitals, federal medical centres, state specialist hospitals as well as a few private hospitals. These are also the facilities where most mental health specialists practice and are mostly located within urban settings [7].

### The mental health care framework in Nigeria

Nigeria has a high burden of mental health conditions, with associated limited availability, accessibility, and affordability of mental health care services [3]. The trained mental health care providers treat a wide range of cases, though they are majorly complex. With a population of more than 200 million people [8,9], and less than 300 psychiatrists who are mostly urban-based, there exists a ratio of 1:700,000 regarding psychiatrists to the entire population of the country is 1:700,000 [10]. Hence, most patients do not have access to mental health specialists who are mainly available at tertiary health facilities.

Fadele et al. identified a considerable treatment gap for prevalent mental health diseases in Nigeria, such as depression and anxiety disorders [9].According to the study, more than 80% of people with serious mental and neurological difficulties are hesitant to use mental health institutions, preferring alternative sources of care such as spiritualists or herbalists before pursuing conventional mental health treatment [9].The study also indicated that the scarcity of mental health experts contributes to the underutilization of mental health care among those with serious mental illnesses, such as schizophrenia and bipolar disorder [9].

Mental health conditions have a high negative impact on the economy and constitute a great economic burden [11]. Low productivity and job losses are common mental health consequences. People experiencing MHC are at higher risk of extreme poverty, since they are prone to work absenteeism and job loss. The mean total direct cost of US$23.1 was the cost of treatment for an acute phase of an MHC and a single follow-up clinic visit [11]. Both direct and indirect costs were high among people undergoing MHC treatment in Nigeria [11]. Because most people paying out-of-pocket, they are therefore prone to catastrophic health spending [11].

Furthermore, of all the medical specialties available in Nigeria, it is only Family Medicine and Internal Medicine trainees who undergo mostly two-month postings in mental healthcare as part of their own training – a period of training exposure that is grossly inadequate for independent mental health care provision. Therefore, mental health care provision for those in need is limited in terms of accessibility and availability in Nigeria.

### Mental health policies in Nigeria

The Lunacy Ordinance, which was enacted in 1916, was the earliest known mental health law in Nigeria. In 1958, it was later renamed the Lunacy Act in 1958, under which power was given to magistrates and doctors to take custody of people affected by mental illnesses [10]. In 1991, the first mental health gap policy was established, but it was not consolidated with provisions for widespread stressful periods like epidemics when many people can develop mental health conditions on a large scale [7]. Historically, based on the emergence of evidence and recommendations by the WHO, Nigeria’s Mental Health Policy was adopted in 1991 with its implementation delegated to local government authorities [12]. The policy was improperly implemented because of the lack of political will, insufficient funding, and inadequate training and coordination of PHC workers [13]. Furthermore, the revised edition of the policy in 2013, the “Policy on Mental Health Service Delivery”, ultimately advocated for sufficient training, retraining, and ongoing professional education for essential medical professionals regarding the screening, identification, and treatment of MNS conditions across all levels of care [14,15]. Developing novel policies to improve access to adequate care for MNS problems in LMICs, such as Nigeria, aligns with the WHO Sustainable Development Goals [15]; nevertheless, the difficulty is to develop acceptable, effective, feasible, and sustainable strategies.

### Integration of mental health care into primary care

Research has shown that mental health services (MHS) may be efficiently delivered to patients in PHC settings, and if identified, the majority of mental problems can be managed at a low cost [16]. However, available evidence that the integration of mental health treatment into the PHC system is extremely slow [17] and that MHS provided to patients at the PHC appears to be negligible [18,19]. As of 2022, only three Nigerian states (Lagos, Ogun, and Osun) had implemented state-wide mental health training for primary health care workers, who serve as the first point of contact for people suspected of having mental illnesses [20–22]. Therefore, the accomplishments in those few states be extended to other states of the federation for the greater benefit of the citizens at large. Study objectives removed.

### Mental health financing in Nigeria

The financing of mental health services is integrated within Nigeria’s overarching health financing structure. The nation’s healthcare sector and services are funded through public sector taxes, government health insurance, social health insurance, and out-of-pocket expenditures by the majority of the population. The federal government funds the majority of the budget for mental health care services, which ranges between 3.3% and 4.0%, with more than 90% going to the country’s few neuropsychiatrist health facilities [6,23]. Nigeria has virtually no additional special funding options for mental health care outside of these hospitals, particularly at the secondary and tertiary levels of healthcare. As a result, most patients and their families are left to pay for the care they receive.

There is a scarcity of mental health care specialists in Nigeria, and most individuals with mental health conditions are unable to pay for mental health services [24]. Family physicians are contemplating the substantial integration of consolidated mental health services into primary care to augment the patient population receiving mental health care. This study seeks to evaluate the present state of mental health services in Nigeria, leveraging the insights and experiences of family physicians, with an emphasis on identifying the existing gaps in mental health service delivery, especially within primary care settings.

## Materials and methods

### Study design

This qualitative study used an exploratory situational analysis research approach that adopted a qualitative semi-structured interview conducted using an in-depth interview guide in order to understand the complex interactions between the social, cultural, and political factors influencing the current status of mental health service provision in Nigeria. This study utilized a grounded theory research method that is well-suited for understanding the multi-layered challenges and enabling factors surrounding service provision among family physicians who render health care services to mental health patients across all the regions of Nigeria. Data collection was carried out between July 1st, 2023 and January 31st, 2024.

### Study population

The study was conducted among family physicians across the geopolitical zones in Nigeria, where they are domiciled across all the states of the country and treat undifferentiated patients across all age groups and diseases. They also interact with other physicians in different disciplines, including mental health physicians. Thus, they are in a strategic position to give their perception of the situatiocountry’s mental health services.res of the family physicians recruited were those who were either consultants or senior registrars who were in active service. The exclusion criteria were sick family physicians and retired family physicians.

The study exclusively recruited family physicians as participants due to their capacity to offer insights into mental health and its potential integration into primary care settings. As primary healthcare caregivers for patients across all age groups and diseases, including mental health disorders, family physicians possess comprehensive understanding of systemic gaps and challenges. Their vast distribution across various levels of care (primary, secondary, and tertiary) and geographic areas makes them suitable for providing insights on the country’s mental health state.

### Study site

Family physicians reside in all the states in the country where they practice at health facilities at different health care levels. They work at different health facilities where they caprovide care toatients who seek care within the primary care domain. A virtual/on-phone location was used for data collection during the interview.

### Sampling size

A total of 24 participants were purposefully recruited using an a priori sampling estimation method from Chu et al. [25]. Despite the priori sample estimaa prioridata saturation criteria (which is the most common form of sampling estimate for qualitative research) was adopted and guided our final sample size estimation using the principle of thematic saturation, when we reach a point at which additional data no longer yielded new insights. Initial estimates regarding the number of participants were derived based on anticipated heterogeneity inparticipant backgrounds and the feasibility of reaching diverse groups within available time and resource constraints. Our sampling approach was purposive, targeting participants with specific experiences and knowledge relevant to the study objectives. This approach enabled us to capture a broad range of perspectives from the interviews conducted with family physicians across the 6 geopolitical zones.

### Study procedure

Family physicians were selected using a stratified sample technique. Two (2) health facilities were selected by simple random sampling from a list of health facilities in each of the country’s six (6) geopolitical zones. Two (2) interviewees were chosen from the list of family physicians provided by the heads of the Family Medicine units of the two selected facilities by uusingurposeful sampling method. The selected participants were contacted via telephone, and consenting individuals were invited for virtual in-depth interviews.

### Data collection

A male and female research team, comprising experienced qualitative researchers specializing in family medicine and public health, used semi-structured interviews to collect data from the participants, using an in-depth interview guide designed from a literature review and programmatic needs. The participants determined the interview day, time, and venue. After a brief courtesy call, the interviewers warmly welcomed the participants and went over the purpose and methods of the study with them before the interview.The instrument consisted of the participant’s socio-demographic data, their view of the current state of mental health care in Nigeria, factors affecting MHS, facilitating and barriers, how they felt that MHS can be enhanced, and how they felt that MH care could be fully integrated into primary health care in Nigeria.

Participants were contacted by telephone/ mobile phone calls, and a virtual in-depth interview was conducted for each consenting participant after information was provided and consent was sought. The interviews, which lasted an average of 45 minutes, were conducted in English language. During the interviews, only audio and audio-video data were collected. These data were saved on secure, password-protected phones immediately after each interview. The recordings were later transcribed verbatim, backed up on an external drive, and anonymized for analysis.

The principle of data saturation guided data collection, which ensured that data collection continued until no new themes or insights were ememergedhe analysis. The priori was adopted from the study by Chu et al. on “Integrating Mental Health into Primary Care: evaluation of the Health Action for Psychiatric Problems in Nigeria” [25].

Probes were used for clarification and to obtain in-depth information as required. The interview guide was piloted at six healthcare facilities, one from each of Nigeria’s six geopolitical zones. Although the guide was administered across all six facilities, only three participantmetet the inclusion criteria and consented to participate in the pilot. Their feedback was instrumental in refining and improving the guide prior to its use in the main study.

To ensure methodological rigour, the following steps were taken: The credibility of this study was ensured through sampling, piloting, and member checking. Transferability was ensured by providing a clear description of the participants’ characteristics, settings of study settings,logy. The transcriptions were also stored as references. Dependability was ensured through detailed descriptions of the methodologies to enable others to repeat the study if needed.

### Data analysis

A descriptive analysis was used for the socio-demographic dafterwing cleaning and entry into SPSS version 22. The distribution of variables was represented in frequency and percentage.The qualitative data adopted a participatory action research (PAR) design where family physicians and the interviewer actively collaborated at all stages of the interview. While the study was not completely co-designed with all the participants from inception, we integrated participatory principles in some key ways by including some of the participants (experienced family physicians) in refining the interview guide during pilot testing exercises and by sharing the preliminary findings with a subset of participants and community stakeholders through feedback sessions. These sessions helped in providing valuable opportunities for these participants to validate, contest, and elaborate on the interpretations of their narratives, thereby contributing to the co-construction of knowledge, while also ensuring that the findings aimed at informing community-driven strategies by centering participants’ voices in the analysis and recommendations.

Recorded interviews were transcribed verbatim and typeset for easy analysis. Transcripts were reviewed against audio files, seand clarifications were sought fromarticipants when necessary. An adequate data storage plan was adopted by ensuring that the transcribed data were backed up on an external drive for future use for at least 5 years. The study applied content analysis, with the results presented thematically. Codes were generated from the transcripts, progressively organized into categories, and then merged into overarching themes. Two independent coders manually analyzed the data (without software). A codebook was iteratively developed based on initial transcripts, refined through coder discussions, and validated during consensus meetings with the broader research team. Responses were coded and entered into a spreadsheet, and two data coders examined the relationships within the data. New themes and categories were added progressively from the recordings until all transcripts were analysed. Conclusions were drawn to depict the participants’ perspectives, with all the researchers meeting to ensure agreement on the representation of views. We followed the Consolidated Criteria for Reporting Qualitative Research (COREQ) guidelines (S1 COREQ Checklist)

### Ethical considerations

Ethical approval was obtained from the National Health Research Ethics Committee, Nigeria, in compliance with established guidelines for research involving human participants. Approval was also sought from the Management of the selected training centres. All the participarticipantsded with comprehensive information sheets where the study purpose and scope, as well as the characteristics that made them eligible for the study, were detailed. Written informed consent was obtained from all participants before participation. Participants were also assured that their participation was voluntary, with the freedom to withdraw at any point without consequence. Confidentiality was ensured throughout the study period as all the recordings were password-protected, and only the researchers had access, and anonymity was maintained with the use of codes and pseudonyms.

## Results

### Socio-demographic characteristics of the respondents

The sociodemographic information provided by the interviewees includes sex, age, marital status, work setting, and job cadre of interviewees. The participants were fairly balanced by sex, with just over half being male. Most participants were in their 40s, and the majority (87%) were married. Nearly three-quarters practiced in tertiary health facilities, with consultants making up about 60% of the sample (Table 1).

**Table 1:** Demographic characteristics of the interview respondents.

Variable	N = 23	%
**Sex**		
Female	11	47.8
Male	12	52.2
**Age group**		
31-40	6	26.1
41-50	8	34.8
> 51	6	26.1
Not Indicated	3	13.0
**Marital Status**		
Single	3	13.0
Married	20	87.0
**Work Setting**		
Primary	2	8.7
Secondary	4	17.4
Tertiary	17	73.9
**Job Cadre**		
Senior Resident	9	39.1
Consultant	14	60.9

**Fig 1:**
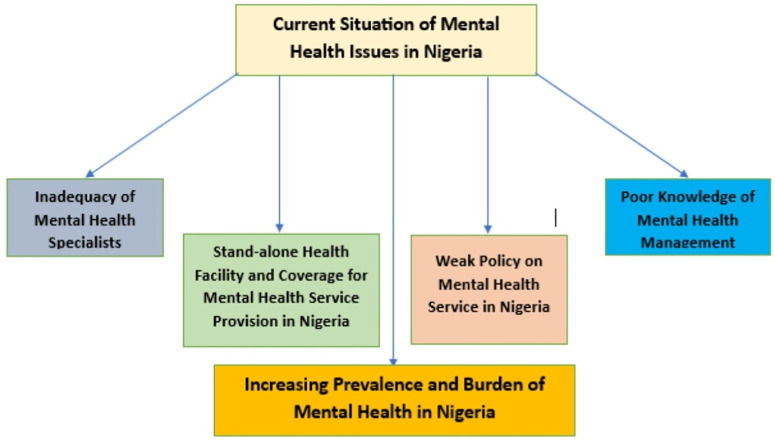
Schematic representation of the current situation of mental issues in Nigeria (Source: Interview Data).

This section outlines the results of in-depth interviews on the current situation omental health (MH) conditions in primary healthcare in Nigeria and the MHNigeria. The responses include a shortage of MH specialists, rising prevalence of mental health conditions, poor awareness or insufficient knowledge of MH management, increasing prevalence and burden of mental health conditions, the stand-alone nature of mental health facilities, and low coverage of mental health services, as shown in Fig 1).

### Structure and coverage of stand-alone health facilities respondents

expressed concerns about the separation of MH services from mainstream healthcare, leading to stigma and reduced access to care.

*“Well, my understanding of the current situation is that mental health care services are structured in such a way that it is totally detached from the mainstream of health care. You know, you have some mental health facilities standing alone. And the problem with that is that people may not really want to be associated with such centres. So, the problem is not putting them as part of the routine.”* Male, Consultant, Ogun State.

A few respondents noted that access to MH care is limited, particularly in states with fewer tertiary institutions and specialized facilities.

*“…sure, that there are not many states that can boast of maybe more than three tertiary institutions across board; so, it means that where the majority of the population reside, mental health care facilities are not available, so the state of mental health facilities is not particularly really good, sincerely.”* (Male, Senior Registrar, Oyo State)

### Inadequacy of Mental Health Specialists

A significant number of respondents highlighted the shortage of MH specialists. Many indicated that the number of specialists is insufficient to meet the growing needs of mental health care across the country. One respondent remarked:

*“They are inadequate. They are far from adequate. Normally, if we look at most of our hospitals, we have several mental health conditions and a few psychiatrists. If we want to consider the prevalence of mental illness in Nigeria as a whole, I think the... I don’t know... the number of psychiatrists that we have in Nigeria is grossly inadequate. Therefore, they actually need the hands of primary care personnel to help them meet these needs.”* (Male, Consultant, Kano State)

***Increasing Prevalence and Burden of Mental Health***: Several respondents pointed out the rising prevalence of mental health conditions, such as schizophrenia, psychosis, and substance abuse, with expectations of a continued increase in the future.

*“Well, the current situation is really not encouraging. I said that because in a few years to come, we might have a lot of people with mental health problems, and it will be so much that even the mental health institutions will be overwhelmed. The reason why I am so bothered is that many people have drug issues and addiction. Yet, the country is not really looking at it as something they need to be proactive about; a lot of youth indulge in illicit drugs. We have people who are into alcohol. We have issues with alcoholism.”-* (Male, Specialist Registrar, Oyo state).

Some respondents noted an increase in suicide rates linked to untreated mental health conditions:

*“The rate of suicide in Nigeria is rising, and then, of course, most of these are due to mental health issues; so, I feel that mental health specialists would the needed for collaboration, to see why we need to work together, you know, to better enhance care.”* (Female, Consultant, Ogun state)

### Poor knowledge of mental health management

There is a notable gap exists in the knowledge of MH management, particularly among primary care physicians, leading to poor diagnosis and treatment.

*“…I think there is a knowledge gap in the primary care system because a recent study conducted in Ghana found that the majority of patients with depression were diagnosed in the primary care system. But because they did not have the appropriate skills, the primary care physicians were unable to actually identify many. So, because of that, there is a need for higher levels of supervision, increased awareness, and also there is a need for primary care physicians to be aware of their role in mental health care”* (Male, Consultant, Kano State)

Respondents mentioned limited awareness of MH conditions, particularly among the lower socioeconomic class. Although discussions on social media have raised awareness, access remains limited to more affluent groups.

*“I think presently, there is not so much awareness about mental health, especially amongst the people of the lower socioeconomic class. I know that quite a lot of discussion is coming up on social media about people’s mental health and all that. But, of course, that is available to people who can access such fora, but for most people who are in the low socioeconomic class, and that is the category that I feel that a majority of Nigerians fall into. Awareness of mental health and its application is very low.”* (Female, Consultant, Ogun state)

### The mental health policy in Nigeria

Respondents highlighted the challenges in implementing Nigeria’s 1991 mental health policy, particularly the lack of awareness among family physicians. One consultant noted that insufficient knowledge of the policy hampthe effectivetive integration of mental health services into primary care. Another study reported inconsistent use of screening tools and training, which affects the management of common mental health conditions and the referral process.

*“The mental health policy adopted in Nigeria in 1991 aimed to integrate mental health services into primary health care. However, many family physicians are still not fully aware of these policies, whhamperingur ability to implement them effectively in our practices.” (Male, consultant, Edo state)*

*“I remember that screening for common diseases at the primary care level is an issue. I cannot technically remember the details, but I know there was routine screening in primary care visits, the use of these evidence-based assessment tools, the regular training for primary care providers on common mental health disorders on their management and referral processes. Also, the issue of health education for both patients and their families……” (Female, Consultant, Sokoto state)*

*“One of the key aspects of the mental health policy is the emphasis on community-based care. Family physicians are often the first point of contact for patients, and we must be equipped with the knowledge and resources outlined in these policies to provide adequate support” (Male, Consultant, Oyo state)*

*“The mental health policy also highlights the importance of public awareness campaigns.” (Male, consultant, Edo state)*

## Discussion

This study is one of the few that focus on family physicians’ perspectives and practices on mental health in Nigeria.The study captures their opinions in a unique way, reflecting both their frontline experience and their potential role in mental health integration in primary care settings.The findings of this qualitative study offer insight into the current situation of mental health services in Nigeria based on the perspectives and experiences of family physicians. The study revealed that the present mental health services in Nigeria is characterized by shortage of mental health specialists, escalating prevalence of mental health conditions, low awareness or insufficient knowledge of the management of mental health conditions, increasing prevalence and burden of mental health conditions, the isolated nature of mental health facilities, ineffective mental health polici, andnd poor access to mental health services. The study shows that family physicians are not only aware of mental health issues but also in a strategic position to support mental health integration programmess.With the right support, they can act as integration catalysts, bridging the gap between community needs and specialist services.

Remarkably, this study found that the inadequacy in the number of mental health specialists is affecting the MHS state in Nigeria. This is corroborated by the report from the Lancet that the number of mental health specialists in Nigeria is grossly inadequate, with fewer than 300 psychiatrists for a population of 200 million individuals [10]. Moreover, a Ryant al also found inadequate mental health specialists in a state in Nigeria, with all the specialists only present in a tertiary facility within an urban setting [26]. The findings of insufficient MH specialists could reflect the low interest of health professionals in mental health speciality in Nigeria.

This study revealed that limited awareness of available mental health services is a significant contributing factor to the current state of mental health in Nigeria. This finding is similar to the report of under-utilization of mental health facilities by Kukoyi et al that the majority of people having severe mental health conditions exhibit reluctance to seek treatment at mental health facilities [27]. Moreover, Lasebikan et al. reported that a large proportion of people suffering from mental health conditions in Nigeria usually seek alternative forms of care with herbalists or spiritualist beforeo attending a mental health facility when they are unsatisfied with the first form of care [28]. In addition, raising awareness of mental health in communities as a component of health programs can enhance MHS use by people [29]. The lack of awareness of the availability of MH services by communities could be attributable to the general belief that MH conditions are addressed at traditional healing centres, thus, resulting in a loss of interest in attempts to search for orthodox health facilities or seek care at such facilities when found.

In this study, poor knowledge of MH conditions and their management was found as a factor deterring the optimization of mental health care. Thisfinding is similar to that ofm Aluh et al which reported a low level of mental health literacy among adolescents in schools [30]. In addition, this index study used healthcare providers as the study population. Therefore, finding that there is a low level of awareness/literacy among the people in the community from both the caregivers and care receivers is a strong indication of this factor in Nigeria’s mental health situation. Furthermore, Bamgboye et al reported a lack of effective training resources for mental health knowledge among health students, where mental health knowledge was absent from the curriculum of some health science students [31]. In a recent study in Nigeria, many family physicians indicated a lack of familiarity or experience with the mental health treatment tool, specifically the use of the WHO guide in mental health treatment [32]. This is also supportive of the lack of knowledge of health professionals regarding mental health in the country.

The participants in this study stated an increasing prevalence of MH conditions. This finding aligns with Patel et al.’s report that mental health conditions are rising globally [33]. The WHO estimated that 20% of people in Nigeria were affected by mental health conditions in the year 2017 [34]. No evidence (due to lack of data) to indicate a rising prevalence of MH conditions over time. However, reports from the Nigeria Bureau of Statistics revealed a steady decline in the prevalence of suicide only [35]. These inconsistent findings may be due to the lack of uniform and general data for the entire country from the different sources.

A low level of coverage of populations with available health facilities was found in this study as the participants noted sparse distribution of mental health facilities and professionals among a large population. This is similar to the report by Fadele et al that there was low coverage of populations in communities regarding the availability of mental health facilities [9]. Furthermore, Abdulmalik et al also reported that there was inadequate coverage of mental health facilities among communities in Nigeria [36]. Ryan et al found a poor distribution of MH specialists in a state in Nigeria with all the specialists only present in a tertiary facility within an urban setting [14]. This congruence of non-uniform distribution of MH facilities could be due to weak planning for the provision of mental health care for populations in the country. The detachment of many MH facilities from the core healthcare facilities was noted in this study. This finding is supported by the reports regarding the non-uniform framework for MH care services in Nigeria, such as the high level of unmet needs by MH patients [27], notable regional variations in the utilization of MH services [37], and poor health care seeking behaviour towards MH care [26]. These findings appear to reflect the different structures for the provision and seeking of MH services in Nigeria.

This study found that the poor implementation of the few available mental health policies in Nigcontributes to thery to inadequate provision of mental healthcare services. This agrees with the findings by Wada et al of the low regard for implementing the policies on mental health care in the country. Other studies have also emphasized the need to restructure and implement Nigeria’s existing mental health policies [38,39].

## Conclusions

This qualitative study engaged family physicians across the six geopolitical zones of Nigeria, who are at the frontline of care for undifferentiated patients. Their perspectives provide crucial insights into the state of mental health services. The results of the data analysis reveal a variety of factors—as perceived by these key providers—that influence mental health care in Nigeria. The findings of this study suggest that a strategic re-evaluation focused on the identified factors is necessary to improve mental health care services. However, it is important to note that these findings are based on the professional observations and experiences of the participating family physicians. While these perceptions are invaluable for understanding systemic challenges, they should be interpreted as such. A more comprehensive and balanced assessment of issues like population-wide mental health literacy would benefit from being triangulated with direct community surveys and quantitative national data. To truly improve mental health care services in Nigeria, all relevant stakeholders must be included in a process that takes into account these factors and builds upon them through national research. Future efforts must be informed by a combination of healthcare worker perspectives, patient experiences, and robust empirical evidence to ensure they are holistic, effective, and equitable.

## Supporting information

S1 Checklist S1_COREQ_Checklist. (DOCX)
